# The Association between Type 2 Diabetes Mellitus and Thyroid Cancer

**DOI:** 10.1155/2017/5850879

**Published:** 2017-07-09

**Authors:** Young-Gyun Seo, Ho-Chun Choi, Ah Reum An, Do Joon Park, Young Joo Park, Kyu Eun Lee, Sue K. Park, Yunji Hwang, Belong Cho

**Affiliations:** ^1^Department of Family Medicine, Hallym University Sacred Heart Hospital, Anyang, Gyeonggi-do 14068, Republic of Korea; ^2^Department of Family Medicine, Healthcare System Gangnam Center, Seoul National University Hospital, Seoul 03080, Republic of Korea; ^3^Department of Family Medicine, Center for Health Promotion and Optimal Aging, Health Promotion Center for Cancer Survivor, Seoul National University Hospital, Seoul 03080, Republic of Korea; ^4^Department of Internal Medicine, Seoul National University College of Medicine, Seoul 03080, Republic of Korea; ^5^Department of Surgery, Seoul National University Hospital & College of Medicine, Seoul 03080, Republic of Korea; ^6^Department of Preventive Medicine, Seoul National University College of Medicine, Seoul 03080, Republic of Korea; ^7^Department of Biomedical Science, Seoul National University Graduate School, Seoul 03080, Republic of Korea; ^8^Cancer Research Institute, Seoul National University, Seoul 03080, Republic of Korea; ^9^Advanced Institutes of Convergence Technology, Seoul National University, Suwon, Gyeonggi-do 16229, Republic of Korea; ^10^Institute on Aging, Seoul National University College of Medicine, Seoul 03080, Republic of Korea

## Abstract

**Aim:**

The incidence of thyroid cancer is increasing worldwide. The prevalence of type 2 diabetes mellitus (T2DM) is also increasing. Therefore, we aimed to analyze the effect of T2DM on thyroid cancer.

**Methods:**

A case-control study was performed. A total of 415 healthy controls with thyroid ultrasound screening and physician consultation were selected from the Thyroid Cancer Longitudinal Study (T-CALOS). Among patients with thyroid cancer who were enrolled in T-CALOS, 415 patients were matched to the control group according to age and sex. We assessed the effects of T2DM, T2DM duration, and T2DM medication on thyroid cancer.

**Results:**

Women with T2DM had lower odds of thyroid cancer than women without T2DM (odds ratio [OR]: 0.40, 95% confidence interval [CI]: 0.20–0.81). Individuals receiving T2DM medication had higher odds of thyroid cancer compared to those without T2DM medication (OR: 5.21, 95% CI: 1.58–17.15). Individuals with T2DM duration <6 years had lower odds of thyroid cancer compared to those without T2DM (OR: 0.58, 95% CI: 0.34–0.97).

**Conclusions:**

Individuals with early T2DM are presumed to have a low incidence of thyroid cancer, and this effect seems to last up to 6 years after diagnosis of T2DM.

## 1. Introduction

The incidence of thyroid cancer is increasing worldwide. The increase in the incidence of thyroid cancer is likely due to a combination of the increase due to more sensitive diagnostic procedures and the true increase due to increased exposure to radiation and to other yet unknown carcinogens [1, 2]. Because of a particularly significant increase in Korea [3], the Thyroid Cancer Longitudinal Study (T-CALOS) was initiated in 2010 to analyze the characteristics of thyroid cancer [4].

The incidence and prevalence of type 2 diabetes mellitus (T2DM) vary from country to country, but the overall trend is increasing prevalence of T2DM in all countries [5]. The prevalence of diabetes among adults 30 years or older is 13.7% and more than 30% of persons aged ≥65 years have diabetes in Korea in 2014 [6].

The association between T2DM and several types of cancers is well known [7–11], but an association between T2DM and the incidence of thyroid cancer is still controversial. A prospective cohort study found that women with T2DM had an increased risk of thyroid cancer [12]. However, a pooled analysis demonstrated that T2DM was not associated with an increased risk of thyroid cancer [13], and a previous literature review reported that the findings are controversial and that any association between T2DM and thyroid cancer was probably weak [14]. Finally, a recent meta-analysis concluded that T2DM conveyed an increased risk of thyroid cancer in women [15].

With regard to the effects of T2DM duration on thyroid cancer risk, a previous study reported no overall association between T2DM and thyroid cancer, but patients with T2DM duration <5 years had a significantly lower risk [16]. With regard to the possible effects of T2DM medications on the incidence of thyroid cancer, recent studies have demonstrated that metformin facilitates thyroid cancer cell apoptosis [17] and that metformin use in patients with T2DM may reduce the risk of thyroid cancer [18].

While several studies have analyzed the effects of T2DM and T2DM medication on thyroid cancer, studies analyzing those effects in the current environment of a rapidly increasing incidence of thyroid cancer are rare. To address this, we analyzed data from T-CALOS to study the effects of T2DM, T2DM duration, and T2DM medication on thyroid cancer in this current environment.

## 2. Materials and Methods

### 2.1. Study Subjects

T-CALOS is an ongoing project undertaken at three general hospitals, Seoul National University Hospital, Seoul National University Bundang Hospital, and National Medical Center, Korea. Thyroid cancer patients and healthy examinees with a normal thyroid confirmed by sonography have been enrolled in T-CALOS. The overall objective of the T-CALOS was to identify the risk factors of thyroid cancer and to evaluate effectiveness of early detection and treatment. Previously, the protocol of T-CALOS was published [4]. The entire study protocol was approved by the Institutional Review Board (IRB) of Seoul National University Hospital (IRB numbers: C-0809-097-258, C-1001-067-307 and C-1202-088-398), the IRB of Seoul National University Bundang Hospital (B-1304/200-401), and the IRB of National Medical Center (H-1308/033-005). All procedures performed were in accordance with the 1964 Helsinki Declaration and its later amendments or comparable ethical standards. Signed informed consent was obtained from all subjects.

**Figure 1:**
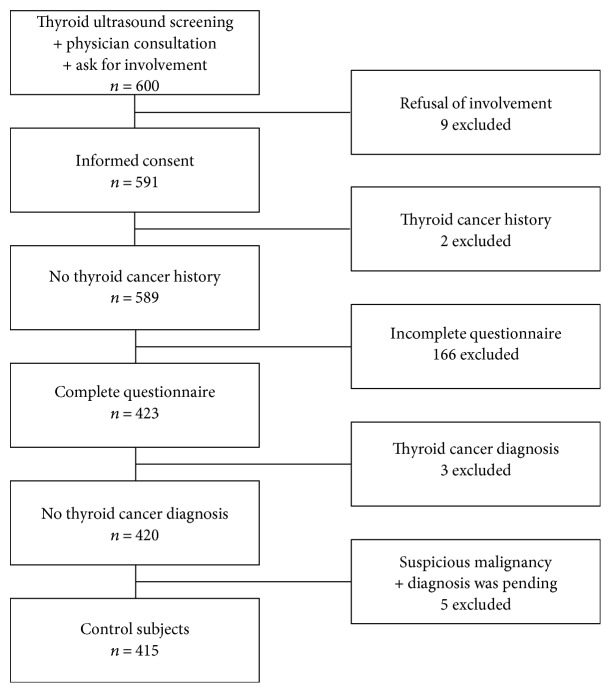
Control population flowchart. Study population and data collection algorithm of control group.

From among healthy examinees who underwent thyroid ultrasound screening and physician consultation between April 2010 and December 2015 at Seoul National University Hospital Center for Health Promotion and Optimal Aging, we recruited about 100 people each year. A total of 591 subjects agreed to enroll in T-CALOS. We excluded subjects who had a history of thyroid cancer or were unable to complete the study questionnaire. Furthermore, we excluded subjects whose thyroid sonographic findings were suspicious for malignancy and those subjects with a pending diagnosis or who had been diagnosed with thyroid cancer by fine-needle aspiration cytology. Finally, we excluded the examinees whose records for T2DM were absent. As a result, 415 subjects were selected as control subjects (Figure 1).

Patients were enrolled in T-CALOS at the time of diagnosis of thyroid cancer. From among 2278 thyroid cancer patients enrolled in T-CALOS between April 2010 and December 2015, 2179 papillary thyroid carcinoma (PTC) patients were included and 415 case subjects were matched to control subjects by age (plus or minus 2 years) and sex.

### 2.2. Definition of Variables

The World Health Organization proposed body mass index (BMI) cutoff point of 25 kg/m^2^ for obesity in adult Asians [19]. So, we defined obesity as a BMI ≥25 kg/m^2^.

Data on the following variables were collected at enrollment: age, sex (male or female), average monthly household income (<3 million KRW, 3–6 million KRW, or ≥6 million KRW), education level (≤elementary school, middle or high school, or ≥college), smoking status (never or ever), alcohol intake (never or ever), regular exercise (no or yes), and obesity (no or yes). A history of T2DM, hypertension (HTN), dyslipidemia (DL), ischemic heart disease (IHD), benign thyroid disease (hyperthyroidism, hypothyroidism, thyroiditis, and goiter), or other cancers (stomach, liver, colorectal, breast, cervical, lung, prostate, urinary bladder, leukemia, lymphoma, and others) was based on a self-reported questionnaire asking if the participants had ever been diagnosed with each disease. So, each disease variable was defined as a self-reported physician's diagnosis of the disease prior to the enrollment of this study. In other words, individuals with a diagnosis of T2DM prior to the diagnosis of thyroid cancer were defined as T2DM patients. Also, T2DM duration (from the age at diagnosis of T2DM to the age at diagnosis of thyroid cancer in the case group and from the age at diagnosis of T2DM to the age at thyroid ultrasound screening in the control group) and T2DM medication (no or yes) were included. There were no insulin users among subjects with T2DM. All the variables mentioned above reflect the status before the completion of the questionnaire. We also measured fasting blood glucose at the time of enrollment by taking a venous blood sample after 12 hours of fasting from all participants. There was no abnormal fasting glucose or impaired fasting glucose among subjects without diabetes.

### 2.3. Statistical Analysis

Student's *t*-test and Pearson's chi-squared test were used for descriptive statistical analyses to compare the PTC cases with their matched healthy controls. Conditional logistic regression models were used to evaluate the effects of T2DM, T2DM duration, and T2DM medication on thyroid cancer. Adjusted odds ratios (ORs) were calculated after adjusting for T2DM duration, T2DM medication, average monthly household income, education level, smoking status, alcohol intake, regular exercise, obesity, HTN, DL, IHD, benign thyroid disease, and other cancers. The confounding variables were selected for each model according to the independent variables. In other words, the T2DM-related variables were included in the case of T2DM patients only, but the T2DM-related variables were excluded when the subjects were both individuals with T2DM and individuals without T2DM. In addition, the receiver operating characteristic (ROC) curve after adjusting for potential confounding factors, criterion based on Youden's Index (*J*), and criterion of the point on the ROC curve closest to the point (0,1) were used to evaluate whether a specific cutoff point of T2DM duration for thyroid cancer risk existed. All statistical analyses were conducted using Stata/MP version 14.0 (StataCorp, College Station, TX, USA). All statistical tests were two-sided and statistical significance was determined at *P* value <0.05.

## 3. Results

### 3.1. Subject Characteristics

The subject characteristics in the control and thyroid cancer groups are shown in Table 1. Because the two groups were matched by age and sex, the mean age in both groups was 55 years and 52% of subjects were women in both groups. Subjects diagnosed with thyroid cancer tended to have a lower average monthly household income (*P* < 0.001), education level (*P* = 0.038), proportion of never alcohol intake (*P* = 0.029), and proportion of DL (*P* = 0.011). Borderline significant differences in the proportion of obesity (*P* = 0.071), T2DM (*P* = 0.090), IHD (*P* = 0.099), and other cancers (*P* = 0.088) were observed, and no significant differences in the proportion of smoking, regular exercise, HTN, and benign thyroid disease were observed.

### 3.2. T2DM Duration and T2DM Medication Use According to the Presence or Absence of Thyroid Cancer

T2DM duration and T2DM medication use according to the presence or absence of thyroid cancer are shown in Table 2. No significant differences between subjects with thyroid cancer and subjects without thyroid cancer in the mean T2DM duration were observed but significant differences in categorical proportions of T2DM duration were observed (*P* = 0.049). Furthermore, the thyroid cancer group tended to have a lower proportion of T2DM duration <5 years when compared to individuals without thyroid cancer after excluding individuals with T2DM duration ≥5 years (*P* = 0.049). Meanwhile, the thyroid cancer group had a higher proportion of subjects who had received T2DM medication when compared to individuals without thyroid cancer (*P* = 0.003).

### 3.3. The Effects of T2DM and T2DM Medication on Thyroid Cancer

The effects of T2DM and T2DM medication use on thyroid cancer are shown in Table 3. The odds of thyroid cancer were lower among individuals with T2DM versus individuals without T2DM, but the differences were of borderline significance (*P* = 0.092). After stratification by sex, the odds of thyroid cancer were lower only in women with T2DM when compared to women without T2DM, even after adjusting for potential confounding factors (OR: 0.40, 95% confidence interval [CI]: 0.20–0.81, *P* = 0.011).

Meanwhile, individuals receiving T2DM medication had higher odds of thyroid cancer compared to those without T2DM medication, even after adjusting for potential confounding factors (OR: 5.21, 95% CI: 1.58–17.15, *P* = 0.007).

### 3.4. The Effects of T2DM Duration on Thyroid Cancer

We derived the odds of thyroid cancer for individuals with T2DM duration ≥5 years with reference to individuals with T2DM duration <5 years (Table 4). No significant differences between individuals with T2DM duration <5 years and individuals with T2DM duration ≥5 years in the odds of thyroid cancer were observed.

We also compared the odds of thyroid cancer among individuals with T2DM duration <5 years and individuals with T2DM duration ≥5 years with reference to individuals without T2DM (Table 4). However, no significant differences in the odds of thyroid cancer were observed among individuals without T2DM, individuals with T2DM duration <5 years, and individuals with T2DM duration ≥5 years.

We additionally compared the odds of thyroid cancer between individuals without T2DM and those within each category of T2DM duration (Table 5) and found that the negative relationship between early T2DM and thyroid cancer is observed up to 6 years of T2DM duration. If the period is limited to 7 years, the odds for thyroid cancer are increasing as the period of T2DM increases in individual with T2DM duration below a certain period (OR = 0.93, *p* for trend = 0.040). However, if the period is longer than that, *p* for trend is not significant (until 8 years, OR = 0.96, *p* for trend = 0.136; until 9 years, OR = 0.96, *p* for trend = 0.117; until 10 years, OR = 0.97, *p* for trend = 0.213, resp.).

### 3.5. Cutoff Point of T2DM Duration for Thyroid Cancer Risk

**Figure 2:**
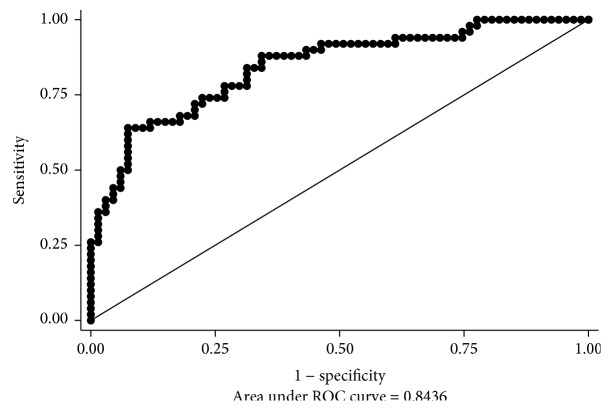
Cutoff points of diabetes duration for thyroid cancer risk. Receiver operating characteristic (ROC) curve of diabetes duration for thyroid cancer risk by logistic regression.

We constructed the ROC curve after adjusting for potential confounding factors to determine if there is a specific cutoff point in the T2DM duration that changes the risk of thyroid cancer (Figure 2). Furthermore, we used a criterion based on Youden's Index (*J*) and a criterion of the point on the ROC curve closest to the point (0, 1) and found that 2 years and 6 years of T2DM duration is the cutoff point for thyroid cancer risk, respectively (sensitivity = 0.64, specificity = 0.93, *J* = 0.57; sensitivity = 0.74, specificity = 0.78, distance between ROC plot and point (0, 1) = 0.34; area under ROC curve = 0.84).

## 4. Discussion

In this study, we analyzed the effects of T2DM, T2DM duration, and T2DM medication on thyroid cancer in this environment of rapidly increasing incidence.

Individuals with T2DM had lower odds of thyroid cancer than those without T2DM, but the observed differences were of borderline significance. After stratification by sex, the odds of thyroid cancer were lower only in women with T2DM when compared to women without T2DM. Our results are contrary to previous studies showing that T2DM was associated with an increased risk of thyroid cancer in women [12, 15]. Also, individuals with early T2DM had lower odds of thyroid cancer than those without T2DM in our study. This is consistent with a previous study and suggests that early T2DM may have a potential protective effect against the development of thyroid cancer [16]. Our results may reflect a higher proportion of subjects with early T2DM compared to previous studies, but no information regarding the duration of T2DM was available in these previous studies [12, 15].

Individuals receiving T2DM medication had higher odds of thyroid cancer compared to those individuals not receiving T2DM medication. This suggests that overall T2DM medications are potential risk factors for thyroid cancer, but the individual effects of each T2DM medication were not evaluated. Previous studies have shown that metformin use in patients with T2DM may reduce the risk of thyroid cancer [18], but our study showed that the use of T2DM medications in patients with T2DM may have higher odds of thyroid cancer. Compared to other studies, the subjects in this study was likely to have a lower rate of metformin use and a higher rate of use of other T2DM drugs. For example, metformin is the most commonly used drug in the recent years, but the use of DPP-4 inhibitors has increased significantly in Korea over the past decade [20]. It is also possible that other T2DM medications except metformin are potential risk factors for thyroid cancer. A previous study reported that sulfonylurea use in patients with T2DM may increase the risk of thyroid cancer [16], but a recent study reported that rosiglitazone use in patients with T2DM may reduce the risk of thyroid cancer [21]. Also, two recent studies found that thyroid cancer is not associated with the use of pioglitazone or insulin [22, 23]. There are various types of T2DM medication including biguanides, sulfonylureas, thiazolidinediones, and insulin. There are also meglitinides, D-phenylalanine derivatives, *α*-glucosidase inhibitors, glucagon-like-peptide-1 agonists, dipeptidyl peptidase-4 inhibitors, and sodium-glucose co-transporter-2 inhibitors, but studies analyzing the effects of these T2DM medications on the incidence of thyroid cancer are rare. Therefore, we cannot fully determine the effect of T2DM medication on the incidence of thyroid cancer until the individual effect of each T2DM medication is analyzed.

Our results suggest that early T2DM may have a potential protective effect against thyroid cancer. Previous studies have shown that metformin inhibits cell cycle progression and induces apoptosis, leading to an antimitogenic effect and decreased the growth stimulatory effect of insulin [17, 24]. Since metformin is commonly used as the first-line therapy in early T2DM [25], the potential protective effect of early T2DM on thyroid cancer may be explained by the use of metformin, as suggested in a previous study [16]. Additionally, individuals with early T2DM may have more frequently used metformin alone, increasing the potential protective effect of metformin and decreasing the unknown effects of other T2DM medications. Because we found that subjects in this study with early T2DM had lower odds of thyroid cancer, but that our subjects with T2DM medication had higher odds of thyroid cancer than those without T2DM medication, it is difficult to explain the potential protective effect on thyroid cancer in early T2DM by metformin alone. One possibility is that in our study, individuals with early T2DM were diagnosed early in the course of diabetes and started taking metformin quickly, and it is possible that diabetes control is being done well with metformin alone. High level of fasting plasma glucose [26] and presence of insulin resistance [27, 28] are associated with thyroid cancer. Therefore, the possibility that well-controlled T2DM may have a negative relationship with thyroid cancer may be considered. Further evaluation of the potential protective effects of early T2DM on thyroid cancer is needed.

We also demonstrated that the potential cutoff point for thyroid cancer risk was 6 years of T2DM duration. We additionally compared the odds of thyroid cancer between individuals without T2DM and those within each category of T2DM duration and found that the negative relationship between early T2DM and thyroid cancer is preserved to 6 years of T2DM duration. These findings suggest that the potential protective effect on thyroid cancer of early T2DM is likely to change over 6 years.

There are several limitations in this study. This study was based on a questionnaire, and the responses might have been affected by recall bias. In addition, volunteer bias should be considered in this study because healthy examinees were included as a control group. Also, this study may be considered limited in its ability to demonstrate causal relationships for thyroid cancer risk. However, we selected only individuals with a diagnosis of T2DM prior to the diagnosis of thyroid cancer and calculated the T2DM duration from the age at diagnosis of T2DM to the age at diagnosis of thyroid cancer in the case group and also calculated the T2DM duration from the age at diagnosis of T2DM to the age at thyroid sonography in the control group. Therefore, the causal relationship can be explained in terms of time. Finally, the analysis of the effect of T2DM medication on thyroid cancer was limited because of a relatively small number of subjects with T2DM, and analysis of the individual effects of each T2DM medication was impossible because of absent information regarding the type of T2DM medication prescribed.

There are several strengths in this study. For example, we analyzed the effects of T2DM, T2DM duration, and T2DM medication on thyroid cancer in this environment of rapidly increasing incidence. We found that subjects with early T2DM had lower odds of thyroid cancer, a finding consistent with a previous study [16]. We also found that the potential protective effect of early T2DM on thyroid cancer is preserved to 6 years of T2DM duration and the statistical significance is reduced after 6 years of initial diagnosis of T2DM, a finding not previously reported. The lower odds of thyroid cancer in individuals with T2DM, compared to those without T2DM, were observed only in women, which is contrary to a previous study showing an increased risk of thyroid cancer in women with T2DM [12, 15]. Future prospective studies with more individuals with late T2DM are needed to evaluate and compare the thyroid cancer risks associated with T2DM, T2DM duration, and T2DM medication use.

## 5. Conclusions

In conclusion, individuals with early T2DM are presumed to have a low incidence of thyroid cancer, and this effect seems to last up to 6 years after diagnosis of T2DM. It is possible that early detection and well control of diabetes may affect not only management of diabetes but also incidence of thyroid cancer. This study serves as a basis for future prospective studies on thyroid cancer risk assessment and comparison among individuals with chronic disease.

## Figures and Tables

**Table 1:** Subject characteristics.

Characteristics	No thyroid cancer (*n* = 415) Mean ± SD or *N* (%)	Thyroid cancer (*n* = 415) Mean ± SD or *N* (%)	*P* value^a^
Age (years)	54.9 ± 10.2	54.9 ± 10.2	
Sex			
Male	199 (48.0)	199 (48.0)	
Female	216 (52.0)	216 (52.0)	
Income (million KRW)			<0.001
<3	67 (16.1)	120 (28.9)	
3–6	177 (42.7)	186 (44.8)	
≥6	171 (41.2)	109 (26.3)	
Education			0.038
≤Elementary school	17 (4.1)	24 (5.8)	
Middle or high school	155 (37.4)	184 (44.3)	
≥College	243 (58.6)	207 (49.9)	
Smoking			0.515
Never	271 (65.3)	262 (63.1)	
Ever	144 (34.7)	153 (36.9)	
Alcohol			0.029
Never	185 (44.6)	154 (37.1)	
Ever	230 (55.4)	261 (62.9)	
Regular exercise			0.329
No	181 (43.6)	195 (47.0)	
Yes	234 (56.4)	220 (53.0)	
Obesity^b^	116 (28.0)	140 (33.7)	0.071
T2DM	67 (16.1)	50 (12.1)	0.090
Hypertension	112 (27.0)	116 (28.0)	0.756
Dyslipidemia	133 (32.0)	100 (24.1)	0.011
Ischemic heart disease	20 (4.8)	11 (2.7)	0.099
Benign thyroid disease	81 (19.5)	67 (16.1)	0.204
Other cancers	25 (6.0)	38 (9.2)	0.088

SD: standard deviation; T2DM: type 2 diabetes mellitus. ^a^*P* value from a *χ*^2^ test for binary outcomes, comparing differences between any 2 study groups. ^b^Body mass index (weight in kilograms divided by height in meters squared) ≥ 25 kg/m^2^.

**Table 2:** T2DM duration and T2DM medication use according to the presence or absence of thyroid cancer.

	No thyroid cancer (*n* = 415) Mean ± SD or *N* (%)	Thyroid cancer (*n* = 415) Mean ± SD or *N* (%)	*P* value^a^
T2DM duration (years)	5.8 ± 7.9	5.4 ± 4.1	0.720
T2DM duration			0.049
<1 year	18 (26.9)	4 (8.0)	
1–3 years	16 (23.9)	12 (24.0)	
3–5 years	6 (9.0)	9 (18.0)	
≥5 years	27 (40.3)	25 (50.0)	
T2DM duration			0.139
No T2DM	248 (83.9)	365 (88.0)	0.296^b^
<5 years	40 (9.6)	25 (6.0)	0.049^c^
≥5 years	27 (6.5)	25 (6.0)	0.664^d^
T2DM medication			0.003
No	34 (50.8)	12 (24.0)	
Yes	33 (49.3)	38 (76.0)	

SD: standard deviation; T2DM: type 2 diabetes mellitus. ^a^*P* value from a *t*-test for continuous outcomes or *χ*^2^ test for binary outcomes, comparing differences between any 2 study groups. ^b^*P* value from a *χ*^2^ test comparing a difference between T2DM duration <5 years and ≥5 years. ^c^*P* value from a *χ*^2^ test comparing a difference between no T2DM and T2DM duration <5 years. ^d^*P* value from a *χ*^2^ test comparing a difference between no T2DM and T2DM duration ≥5 years.

**Table 3:** The effects of T2DM and T2DM medication on thyroid cancer.

	Adjusted^a^		*P* value	Adjusted^b^		*P* value
	OR	(95% CI)		OR	(95% CI)	
T2DM versus no T2DM						
Total (415 cases/415 controls)	0.71	(0.48–1.06)	0.091	0.70	(0.46–1.06)	0.092
Male (199 cases/199 controls)	0.97	(0.58–1.61)	0.896	1.00	(0.58–1.74)	0.991
Female (216 cases/216 controls)	0.45	(0.23–0.85)	0.014	0.40	(0.20–0.81)	0.011
T2DM medication versus no T2DM medication (50 cases/67 controls)	2.93	(1.27–6.76)	0.011	5.21	(1.58–17.15)	0.007

OR: odds ratio; CI: confidence interval; T2DM: type 2 diabetes mellitus. ^a^Adjusted for matching variables (age and sex). ^b^Adjusted for matching variables (age and sex), average monthly household income, education level, smoking status, alcohol intake, regular exercise, obesity, hypertension, dyslipidemia, ischemic heart disease, benign thyroid disease, and other cancers in T2DM versus no T2DM; adjusted for matching variables (age and sex), T2DM duration, average monthly household income, education level, smoking status, alcohol intake, regular exercise, obesity, hypertension, dyslipidemia, ischemic heart disease, benign thyroid disease, and other cancers in T2DM medication versus no T2DM medication.

**Table 4:** The effects of T2DM duration on thyroid cancer.

	Adjusted^a^		*P* value	Adjusted^b^		*P* value
	OR	(95% CI)		OR	(95% CI)	
T2DM duration (50 cases/67 controls)						
<5 years	Reference			Reference		
≥5 years	1.13	(0.51–2.47)	0.764	0.67	(0.23–1.98)	0.469
T2DM duration (415 cases/415 controls)						
No T2DM	Reference			Reference		
<5 years	0.60	(0.35–1.00)	0.051	0.59	(0.34–1.02)	0.060
≥5 years	0.88	(0.50–1.55)	0.665	0.86	(0.47–1.57)	0.615

OR: odds ratio; CI: confidence interval; T2DM: type 2 diabetes mellitus. ^a^Adjusted for matching variables (age and sex). ^b^Adjusted for matching variables (age and sex), T2DM medication, average monthly household income, education level, smoking status, alcohol intake, regular exercise, obesity, hypertension, dyslipidemia, ischemic heart disease, benign thyroid disease, and other cancers in T2DM duration ≥5 years versus <5 years; adjusted for matching variables (age and sex), average monthly household income, education level, smoking status, alcohol intake, regular exercise, obesity, hypertension, dyslipidemia, ischemic heart disease, benign thyroid disease, and other cancers in T2DM duration ≥5 years and <5 years versus no T2DM.

**Table 5:** The effects of T2DM duration on thyroid cancer (extended).

	*N*	Adjusted^a^		*P* value	Adjusted^b^		*P* value
		OR	(95% CI)		OR	(95% CI)	
T2DM duration							
No T2DM	713	Reference			Reference		
<1 year	22	0.21	(0.07–0.63)	0.005	0.18	(0.06–0.56)	0.003
<2 years	38	0.34	(0.16–0.71)	0.004	0.30	(0.14–0.64)	0.002
<3 years	50	0.45	(0.24–0.83)	0.010	0.43	(0.23–0.81)	0.009
<4 years	57	0.56	(0.32–0.97)	0.039	0.57	(0.32–1.01)	0.056
<5 years	65	0.60	(0.35–1.00)	0.051	0.59	(0.34–1.02)	0.060
<6 years	72	0.57	(0.35–0.94)	0.028	0.58	(0.34–0.97)	0.039
<7 years	77	0.64	(0.40–1.04)	0.070	0.65	(0.39–1.07)	0.091
<8 years	84	0.62	(0.39–0.98)	0.040	0.62	(0.38–1.01)	0.057
<9 years	91	0.72	(0.46–1.11)	0.136	0.73	(0.46–1.16)	0.187
<10 years	94	0.71	(0.46–1.09)	0.117	0.71	(0.45–1.13)	0.152

OR: odds ratio; CI: confidence interval; T2DM: type 2 diabetes mellitus. ^a^Adjusted for matching variables (age and sex). ^b^Adjusted for matching variables (age and sex), average monthly household income, education level, smoking status, alcohol intake, regular exercise, obesity, hypertension, dyslipidemia, ischemic heart disease, benign thyroid disease, and other cancers.
